# Cross-sectional survey on self-reported health of ambulance personnel

**DOI:** 10.1186/s13049-015-0087-1

**Published:** 2015-02-08

**Authors:** Emese Pek, Kata Fuge, Jozsef Marton, Balint Banfai, Gabriella Csaszarne Gombos, Jozsef Betlehem

**Affiliations:** Faculty of Health Sciences, Institute of Emergency Care and Health Pedagogy, University of Pecs, 4 Vorosmarty street, H-7621 Pecs, Hungary; Faculty of Health Sciences, Institute of Sport Sciecnes and Physiotherpy, University of Pecs, 33 Landorhegyi street, H-8900 Zalaegerszeg, Hungary

**Keywords:** Ambulance personnel, Self-reported health status, SF-36, General health

## Abstract

**Background:**

The high job stress among ambulance personnel is a widely known phenomenon. *Purpose*: to asses the self reported health status of ambulance workers.

**Methods:**

An anonym self-fill-in questionnaire applying SF-36 was used among workers from the northern and western regions of Hungarian National Ambulance Service.

**Results:**

Based on the dimensions of the SF-36 questionnaire the respondents considered their “Physical Functioning” the best, while “Vitality” was regarded the worst. The more time an employee have been worked at the HNAS the worse his health was in the first four dimensions like, “*Physical Functioning*”, “*Role*-*Physical*”, “*Bodily Pain*”, “*General Health*”: p < 0.001. Those working in secondary part-time jobs considered their health in all dimensions worse. The respondents who did some kind of sports hold their health in all dimensions better (p < 0.001). The workers with higher BMI regarded their health status worse, in four dimensions: “*Physical Functioning*”: p = 0.001; “*Role*-*Physical*”: p = 0.013; “*General Health*”: p < 0.001; “*Role*-*Emotional*”: p = 0.05.

**Conclusions:**

The workers health status proved to be insufficient according to the subjective perception and measurable parameters. According to the subjective perception of health and measurable parameters of health status of workers proved to be insufficient. Poor physical health can lead indirectly to psychological problems, which may lower the quality of the work and can lead to high turn-over.

## Background

The labour of emergency personnel is in the forefront of ambulance care. Members of the Hungarian National Ambulance Service (HNAS) experience unexpected situations during their day-to-day work, which require prompt action, immediate decision-making ability and exceptionally good reactions. It has been proved already, that these workers, based on the characteristics of their job are exposed to far greater physical and mental pressure than the general population or other medical workers [1-3]. In the light of these findings an increasing amount of literature has been published on the workers of pre-hospital medical service, in Hungary as well. Most separate research of the field examines the causes, consequences and risk factors of the mental exertion, alongside exploring the possible coping mechanisms [4,5].

Systematic mapping of the Hungarian society’s health status has a history of several decades: started in 1984 by the micro-census of the Hungarian Central Statistical Office (HCSO), followed by the same organisation’s Health Status Evaluation in 1994, the Hungarostudy survey carried out three times since 1988 (1988, 1995, 2002), and the National Health Interview Survey (NHIS) in 2000 [6]. Since 2008 the European Parliament and the European Council have been prescribing the examination based on the community survey at the European level – the HCSO completed the first, internationally standardized European Health Interview Survey (EHIS 2009) in Hungary among the population over 14 years at the fall of 2009 accordingly [7]. The questionnaire, which is similar to our own research tool consisted of the present state of health (the comparison is based on the response options with was a 5-point scale), health-state limitations, BMI (same as the WHO categorization), physical activity, smoking and alcohol consumption habits. The findings of EHIS 2009 lead to the conclusion that more than half of the adult population is satisfied with their health status, which shows a declining tendency by the passing of time. 6.8% of the respondents claimed restrictions because of their health status; however, the majority is not limited in the smooth execution of everyday activities even in the case of chronic diseases. Results show a growing tendency of health-consciousness, and primary prevention proves to be increasingly important to the population as the majority of respondents regularly attend screening tests.

To date, several research covers the lifestyle, health status and social conditions of medical workers worldwide [7-9]. Data from the Hungarostudy 2002 form the base of the 2008 publication “Are Medical Workers Healthier?” The research revealed that medical workers evaluated their own health status more satisfying compared to non-medical workers (p = 0.047), and also that the prevalence of the examined diseases was lower among them (musculoskeletal diseases: p = 0.043; heart diseases: p = 0.036) [8]. A 2004 study covering specifically the health status of medical workers dealing with severe cases (patients of oncology and hospice wards) revealed that the incidence of addictions is much greater within the examined population than that of the Hungarian population or other medical workers [9].

The first significant Hungarian study examining specifically the ambulance workers was carried out in 2005, covering only the Southern Great Plain Region [10]. This cross-sectional survey (n = 407) claimed, that 70% of the respondents had body-mass index (BMI) over the normal range. Recognizing the gravity of the problem, a nationwide examination was carried out in Hungary in 2008 to collect more precise and demonstrative data, aiming to map subjective, self-reported health status of the workers at the HNAS [11,12]. Number of participants was 364. Self-destructive habits as a way of relaxation in a stressful life were highly represented within the sample: 415% of the respondents claimed to be a regular smoker. BMI of the study’s respondents fell in the abnormal range in 75% of the cases. Furthermore, the study examined the factors influencing the evaluation of health status in detail, using statements such as regular physical activity improves the self-evaluation of health status. For later comparison, we have taken on board a number of elements with the same response options from researches also based on self-reporting questionnaire (part-time job, BMI, addictions). Focusing on problems rooted in emotional pressure and their overcoming, incidence of posttraumatic stress disorder (PTSD) within emergency care providers was examined first in Budapest (capital of Hungary) in 2008 [4], then nationwide in 2010 [5]. The latter study examined the addictions as a coping mechanism of PTSD as well. Both studies revealed that PTSD is indeed present among members of the HNAS, and the respondents’ gender (p = 0.003) and the experience of extremely tragic cases during work (p = 0.001) also have large influence on the pathomechanism. Medication- and drug usage was reassuringly low among PTSD-affected workers, while the incidence of addiction-related habits showed no relationship to the presence or degree of PTSD either.

Most international scientific literature covering the health status of ambulance workers has been primarily focused on the mental exertion [13-16]. It is well known that the helping (assisting) professions are considered high-risk population for mental illness regarding every part of their daily work. They have to deal with the high degree of stress and traumatic experiences, and processing these experiences have large burden on them [17-19]. A 2004 British study [20] examining the PTSD presents among assistive medical attendants revealed that the prevalence of the disease is higher among ambulance workers than among emergency department members (p < 0.05). From the gender perspective – in line with previous literature findings – the male respondents were dominant in the study (p < 0.005). Incidence of the PTSD is proved to be higher within the assistive medical personnel than within the average population, and prevention and well-timed treatment of the disease is an extremely popular topic of the international scientific literature [21-24]. It is now proved that the risk of physical injuries is exceedingly high in the aforementioned group, thus a considerable amount of literature has been published specifically on the workers’ physical and musculoskeletal status of health. Among them there are also some that specifically examine the development of musculoskeletal complaints in the context of PTSD and suffered trauma [25]. A 2005 study [26] from the University of Pittsburgh demonstrated the back injuries and the risk factors of chronic back pain among ambulance workers. In the same year, the Swedish emergency service personnel were examined in terms of disorders of the neck-shoulder and low-back region [27]. Both studies revealed an approximately 50% incidence-rate of these complains. Another study claim that 76.9% of the Japanese ambulance workers suffer from back- and neck pain [28], while the same ratio in the United States of America is 48% [24]. Self-evaluation of health status is therefore a complex category, which cannot be detached from the use and evaluation of the health dimensions [29].

In light of all the above, the aim of this study is to assess the physical and mental health status of the Hungarian ambulance workers (based on their subjective self-evaluation), using an internationally recognized, standardized and validated, generic assessment tool (SF-36) on a representative sample. Furthermore, we intended to compare our results with the already existing Hungarian and international indicators describing the health status of the general population and the ambulance workers.

## Methods

**Figure 1:**
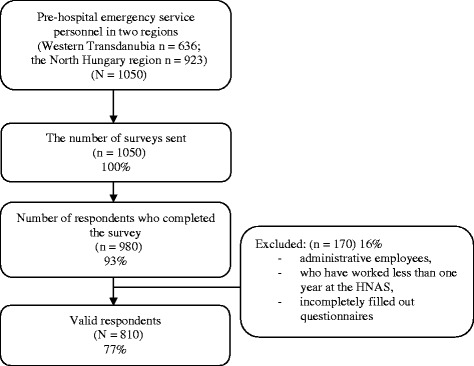
**Flow chart of respondent selection.**

The representative, cross-sectional research work was carried out between October and December 2012, following the collection of all required written permissions (by the Director General and the regional directors of the HNAS), in compliance with the ethical standards, at the ambulance stations of the Western Transdanubia and the North Hungary regions. The study covers the pre-hospital emergency service personnel of 65 ambulance stations in 6 counties of Hungary (Borsod-Abaúj-Zemplén, Heves, Nógrád, Győr-Moson-Sopron, Vas and Zala County). The sample we selected fully covered the Hungarian rescue workers population (both in kind of gender and position) [30]. Apart from applying the ethical restrictions, each participant received information on the substance of the study and was allowed to ask questions regarding the research, all before the completion of the examination. Participants of the study were invited to fill out a questionnaire on a voluntary and anonym basis that consisted of standardized and self-developed questions. Altogether 1050 research tools were sent out by research assistants, who also took part in collecting the questionnaires. 980 pieces were returned, out of which 810 were evaluable. We excluded the incompletely filled questionnaires and those that did not meet the inclusion criteria (administrative employees and those who have worked less than one year at the HNAS) (See Figure 1). An essential part of the research tool used in the current examination was the Short Form-36 Health Survey Questionnaire (SF-36), which was validated in Hungary in 1999 by Ágnes Czimbalmos et. al., and which is based on the functional and perceptional health model [31]. The psychometric tool contains 36 questions, and it is used in more than 50 countries as part of the International Quality of Life Assessment Project (IQOLA). The questions are designed to assess the respondents’ opinion on their own health, by grouping the answers into 8 dimensions [32,33]. These dimensions are the following: *1. Physical Functioning* (PF): limitations in everyday physical activities – 10 items; *2. Role–Physical* (RP): decrease of amount and duration of, completion through difficulties or overall omission of regular activities as a result of physical health – 4 items; *3. Bodily Pain* (BP): pains of the body and their influence on work (at workplace and at home) – 2 items; *4. General Health* (GH): evaluation of personal health, attitudes concerning health status – 5 items; *5. Vitality* (VT): stamina, enthusiasm, weariness, tiredness – 4 items; *6. Social Functioning* (SF): changes and intensity of relationship with friends and family due to physical or emotional problems – 2 items; *7. Role-Emotional* (RE): decrease of amount and duration of, completion through difficulties or overall omission of regular activities as a result of emotional status – 3 items; *8. Mental Health* (MH): nervousness, depression, calmness, peacefulness, sadness and depression, happiness – 5 items. First three elements of the 8 dimensions are assessing mainly the physical status, while the last three elements assess the mental state, with great reliability and validity (80-85%). The fourth and fifth dimensions play a role in both main variables (physical and mental health status) [34]. The last question of the survey form does not belong to the aforementioned dimensions: *Health Modification*: changes in health status compared to the status one year prior – 1 item. Each dimension can take the value of 0 to 100, the higher value representing the more favourable health status. Based on the resulting values it can be claimed, that the higher total score a respondent gains, the healthier he feels and the lower his limitations were. Further questions of the survey form examined the socio-demographic status, working habits, free-time activities, physical state of health and addictions. Resulting data was interpreted by descriptive- (average and frequency calculations) and coherence-revealing mathematical-statistical analysis, by the help of SPSS 20.0 software. Relationship between variables was analysed by Chi-square test, and Mann–Whitney. Kruskal-Wallis test was carried out to prove if the difference or correspondence between variables was significant. Furthermore, correlation analysis and linear regression tests were completed. Results were considered significant in case of p ≤ 0.05.

## Results

40 female and 770 male respondents provided assessable completed survey forms in this study (N = 810): 517 persons from the North Hungary region and 293 persons from the Western Transdanubia region. Average age of respondents was 40 years. Regarding family status, 464 persons were married (57%), 158 respondents were living in a relationship other than marriage (19.5%), 64 persons were divorced (8%), and 2 persons were widowed (0.5%). Majority of respondents were living in a town (499 persons, 61%), 304 persons were living in a village (38%), and 7 persons (1%) claimed to be living in the capital of Hungary. Regarding educational qualifications, majority of respondents held a high school diploma (446 persons, 55%), 183 persons held a degree from a vocational school (23%), 132 persons held a Bachelor degree (16%), 31 persons held a degree from a secondary vocational school without a high school diploma (4%), 11 persons held a university degree (1%) and 7 persons completed only the primary school or less (0.5%). Regarding the level of assignment, 378 respondents (47%) were working as emergency medical technician, 318 respondents as ambulance car drivers (39%) and 104 respondents as paramedics (13%). 10 persons (1.2%) held an MD degree with emergency medical specialization.

Using the *SF-36* questionnaire, the aim of the study was to measure how workers self-assess their health status (see Table 1). Respondents assess their “*Physical Functioning”* to be the best, therefore they don’t feel limitations regarding their everyday activities. “*Vitality” was considered to be the worst*, and they have less enthusiasm and soul energy. Influence of the various scales at each other was compared using the Pearson correlation test. Based on these results it can be claimed, that each dimension is in a statistically proved, significant relationship with the others (p < 0.001) – should a respondent evaluate his status in one dimension better, the health status at all the other dimensions will also be higher. Table 2 presents the influence of the respondents’ *age* on the SF-36 dimensions. As seen in the first four dimensions, the older the respondent is, the worse he would evaluate his own “*Physical Functioning*” (p < 0.001), and the more he perceives the need to decrease the amount or duration of his regular physical activity (“*Role* – *Physical*”: p < 0.001). The older an ambulance worker is, the more bodily pain he perceives (“*Bodily Pain*”: p < 0.001); and the more negative his attitude to health will be (“*General Health*”: p < 0.001). *Regional differences* are not described in this work in detail, since no considerable, significant difference was observed in the reviewed values regarding the two subsamples. Shift work and the large load of physical and mental exertion is a huge burden for all people, and thus they have an influence on health status as well. Weariness, severe tiredness and the feeling of lacking power is common after a 12-hour shift, where the ambulance worker must make decisions affecting peoples’ lives. Supposedly, the longer the ambulance worker has been in his position, the more severe this feeling and status grows. In line of all the above, correlation analysis of the *working years* and the SF-36 scales are shown by Table 3. Based on the verified correlation, the difference is significant in the first four dimensions (describing physical health), as the longer a respondent has been the member of the National Ambulance Service, the worse he evaluates his health status. In light of the tasks carried out during ambulance work, regarding the *level of assignment* it is no surprise that the ambulance drivers evaluate their status of health the most favorable in all 8 dimensions of the SF-36 (they bear the least professional responsibility, thus they have the fewest decisions to make, and it should also be kept in mind that they have the lowest educational background as well). Parallel to this, emergency medical technicians have the lowest scores at almost all dimensions. Not only the number of years served at the Hungarian National Ambulance Service, but also the characteristics of the current work influence the assessment of workers’ health status. In addition to the difficult mental and physical work, many of the workers attend a *secondary part-time job*, which negatively influences the self-assessment of health status by its’ mere existence. Considering the type of the secondary job, those workers seem to evaluate their health status the least favorable, who attends physical and intellectual labour alike. The correlation between the health status assessment and the *number of working hours* (primary and secondary job) is presented in Table 4. Comparing the SF-36 dimensions to the amount of *free time* (hours/week) by linear regression proved significant correlation (p < 0.001), as in the more free time a worker has, the better he evaluates his status of health. *Sport* is known to be a protective factor in maintaining and keeping good health. Apart from positively influencing the bodily health, physical activity is a great opportunity to regenerate intellectually and mentally, stepping out from the everyday buzz, troubles and problems. Regarding sport habits, the significant difference in case of all dimensions was statistically proved (p < 0.001). It can be stated accordingly, that respondents carrying out regular physical activity rate their health better. *Alcohol consumption* proved to have a statistically significant influence on the subjective health assessment based on all SF-36 dimensions – at each dimension, those respondents rated their health status the most favorable, who never drink alcohol (p < 0.001). Respondents who consume alcoholic beverages on a daily basis assess their health the worst, at almost all dimensions. *Smoking* proved to have a significant affect on subjective health assessment in only one dimension. Those respondents have the most positive attitude to health (IV. “*General Health*”), who has never been smoking (87 ± 3) and those consuming even a package a day proved to have the most negative attitude (70 ± 19) (p = 0.003).

**Table 1:** **Self-reported health status of ambulance workers based on the 8 dimensions of SF-36**

**N = 810**		***Abbreviation of scale***	***Min. value***	***n (%)***	***Max. value***	***n (%)***	***Average***	***SD***
**SF-36 scale**	***I. Physical Functioning***	PF	0	2 (0.2)	100	373 (46)	**91**	14
	***II. Role-Physical***	RP	0	43 (5)	100	535 (66)	**83**	29
	***III. Bodily Pain***	BP	0	4 (0.5)	100	376 (46.4)	**82**	22
	***IV. General Health***	GH	0	2 (0.2)	100	17 (2.1)	**64**	20
	***V. Vitality***	V	0	7 (0.9)	100	41 (5.1)	**61**	23
	***VI. Social Functioning***	SF	0	8 (1)	100	370 (45.7)	**80**	24
	***VII. Role-Emotional***	RE	0	67 (8.3)	100	519 (64.1)	**79**	32
	***VIII. Mental Health***	MH	0	8 (1)	100	75 (9.3)	**71**	22

**Table 2:** **Linear Regression between age and SF-36 dimensions among the responding ambulance workers**

***N = 810***		***R***	***Sig. (p)***
***SF-36 scale***	***I. Physical Functioning***	**0.25**	**0 < 001**
	***II. Role-Physical***	**0.14**	**0 < 001**
	***III. Bodily Pain***	**0.15**	**0 < 001**
	***IV. General Health***	**0.2**	**0 < 001**
	***V. Vitality***	0.01	0.59
	***VI. Social Functioning***	0.02	0.45
	***VII. Role-Emotional***	0.03	0.314
	***VIII. Mental Health***	0.001	0.98

**Table 3:** **Influence of age, years served at the HNAS and BMI on self-reported health state among the examined ambulance workers (N = 810)**

***N = 810***		***Labour years (Pearson Correlation)***		***BMI (Pearson Correlation)***	
		***R***	***Sig. (p)***	***R***	***Sig.(p)***
***SF-36 scale***	***I. Physical Functioning***	**−0.2227**	**<0.001**	**−0.112**	**0.001**
	***II. Role-Physical***	**−0.151**	**<0.001**	**−0.087**	**0.013**
	***III. Bodily Pain***	**−0.175**	**<0.001**	−0.027	0.445
	***IV. General Health***	**−0.213**	**<0.001**	**−0.148**	**<0.001**
	***V. Vitality***	−0.023	0.515	−0.043	0.219
	***VI. Social Functioning***	−0.043	0.225	−0.004	0.92
	***VII. Role-Emotional***	−0.069	0.051	**−0.069**	**0.05**
	***VIII. Mental Health***	−0.05	0.157	−0.041	0.239

**Table 4:** **Correlation of the SF-36 dimensions to the type of work and the amount of working hours among ambulance workers**

***SF-36 scale***	***Monthly hours in primary job***	***Monthly hours in secondary job***	***Weekly hours of physical secondary job***	***Weekly hours of intellectual secondary job***	***Weekly hours of medical secondary job***	***Weekly hours of non-medical secondary job***
	***(N = 810)***	***(n = 442)***	***(n = 442)***	***(n = 442)***	***(n = 442)***	***(n = 442) (p=)***
	***(p=)***	***(p=)***	***(p=)***	***(p=)***	***(p=)***	
***I. Physical Functioning***	0.243	0.062	0.206	0.652	0.463	0.056
***II. Role-Physical***	**0.009**	0.455	0.213	0.717	0.283	0.670
***III. Bodily Pain***	0.570	**<0.001**	**<0.001**	0.579	**0.001**	**<0.001**
***IV. General Health***	0.065	**0.005**	**0.002**	0.484	**0.011**	**0.002**
***V. Vitality***	**0.014**	**<0.001**	**0.006**	0.123	**<0.001**	0.059
***VI. Social Functioning***	0.204	**0.001**	**0.029**	0.919	**<0.001**	0.230
***VII. Role-Emotional***	0.122	0.186	0.909	0.762	0.063	0.985
***VIII. Mental Health***	0.213	**<0.001**	**0.040**	0.744	**<0.001**	0.254

Workers with higher *BMI* or those falling in the WHO-category overweight or obese assess their health status worse, resulting from their physical limitations or even from their bodily pains, exhaustion or their deprived social relationships. BMI, as a continuous and a categorical variable, was compared to all eight dimensions of the SF-36 questionnaire based on the subjective health status assessment. Furthermore Table 3 shows the differences between the objectively measured BMI (continuous variable) and the subjective health aspects. The four factors indicated were analyzed by linear regression. In case of all four dimensions a weak negative correlation was observed between the BMI and the health status assessment, as in respondents with higher BMI scores assess their health status more negatively. The higher a worker’s BMI score is, the higher level of daily limitations he is experiencing (“*Physical Functioning”*: p = 0.001), and the more hardship he finds in carrying out everyday activities (“*Role*-*Physical*”: p = 0.013), parallel to which the workers rate their “*General Health”* worse as well (p < 0.001) (Table 3). Furthermore, the higher a worker’s BMI is, the worse he would assess his mental health (“*Role*-*Emotional”*: p = 0.050). On the other hand, BMI categories showed a significant difference in case of only two dimensions. In the dimension of “*Physical Functioning*” (p < 0.001) it can be observed that the subjective assessment of health is the highest when the respondent’s BMI falls into the normal category, while the assessment of „General Health” is the best when the respondent’s BMI falls into the category of normal or slightly underweight (p = 0.017).

## Discussion

The results of the examinations completed and the personal feedbacks both confirm that the research among ambulance personnel described here is extremely timely and necessary. Although rescue workers are a special group of the Hungarian general population (corresponds to the average socioeconomic status), it was definitely important for us to compare the results we obtained to the average Hungarian population data, which are also based on self-reported data. In our sample the ambulance workers assess their health status much better, than those compared to the most prior Hungarian data (EHIS 2009). Table 5 presents the most important comparisons. Self-assessment of the health status demonstrated a decreasing tendency by the increase of age among the current sample (SF-36) as well. Regarding the level of limitations as a consequence of health, 46.8% of the workers of HNAS reported, that they experience obstacles in their everyday activities, compared to the 41.1% rate among the general population. This may be the consequence of the above-average BMI score, which is lower in the average population (58.6%) (male: 60%; female 49%) compared to that of the ambulance workers (75%). It is essential to compare the results of this study to the nationwide research carried out in 2008 among ambulance workers, as the socio-demographic homogeneity (in terms of gender, age, place of residence, level of assignment) of the two samples was approximately similar. The research tools in the two studies were similar in terms of items and response options. Distribution of workers with a secondary job was nearly the same (2008: 58.5%; *2012: 54.6%*). 65% (138 persons) of the respondents completing the survey tool of the first examination were attending physical secondary jobs and 35% (75 persons) were attending intellectual labour. The same rate in the current study is the following: 67% (296 persons) of the respondents attend physical secondary job only, 26% (115 persons) attend intellectual job only, and 7% (31 persons) of the sample attend both physical and intellectual secondary jobs. The negative influence of additional work on health status (the same way as the number of years served at the HNAS) was detectable in the self assessment as well, since it was proved based on one hypotheses of the current study, that ambulance workers with a secondary job assess their health worse according to the SF-36 questionnaire. This correspondence proved to be significant in the 2008 study as well. Regarding addictions, the number of smokers was higher in our study compared to the previous examination’s average (never been smoking or quitted: 2008. 45%; *2012. 33%*). Unfortunately, the rate of those who consume alcohol has also been increasing (2008. 17.9%; *2012. 15%*)*.* The BMI score that measures nutritional status showed a relatively similar value: the majority of the workers (nearly 75%) were above the normal category (BMI > 25 kg/m^2^). This factor influences the “*Physical Functioning*” (p = 0.001), the „*Role*-*Physical*” (p = 0.013), the “*General Health*” (p < 0.001), and the “*Role*-*Emotional*” (p = 0.05) scales negatively based on the SF-36 dimensions. Thus the more chronic (the higher) a worker’s BMI is, the less favorable he assess his status of health. Apart from unhealthy eating, decreased physical activity plays a great role in the development of chronic BMI, which showed extremely good result in the current study compared to the 2008 research. The ratio of those carrying out some kind of sporting activity in the 2 regions of Hungary was 59%, while in 2008 the rate didn’t reach 10% nationwide. Protective role of sports was apparent in the 2008 examination; however, our study revealed that ambulance workers who carry out some kind of physical activity assess their health status more favorable in all dimensions of the SF-36 questionnaire (p < 0.001). In light of the above statements, ambulance workers find their health status to be an obstacle in everyday activities in a lot less instances (2008: 67.6% of respondents find it an obstacle to some extent; 2012: 47%). One conclusion of the current results may be that ambulance worker’s health status based on subjective assessment (SF-36) or objective evaluation (BMI) has been improving greatly since 2008. Unfortunately the rate of chronic BMI and the incidence of addictions are still rather high. It is a reassuring result though, that the proportion of those carrying out sporting activities has been expanding fourfold. Protective and reparative effects of physical activity will hopefully soon be represented by the objective indicators and in the self-assessment as well. Several publications of the international scientific literature cover the self-assessment of health status, some specifically examining ambulance workers. In comparison with a 1991 Irish examination looking at ambulance workers’ BMI, we may state that in the two Hungarian regions currently examined the ratio of BMI-scores above normal was a lot higher (Belfast: 56% above normal range, 10% of them overweight; in the two regions examined in this study: 75% above normal range, approximately 7% of them overweight) [35].

**Table 5:** **Comparison of variables in connection with health status among ambulance workers and the Hungarian population**

**Examined variable**		**Hungarian population**			**Ambulance workers PTE**		
		**(EHIS 2009.)**			**FHSc. 2012.**		
		**(N = 5051)**			**(N = 810)**		
		***Male (%)***	***Female (%)***	***Total (%)***	***Male (%)***	***Female (%)***	***Total (%)***
**Health status**	***Very good, good***	58.9	50	54.2	75.8	87.5	76.5
	***Satisfactory***	29.4	32.7	31.1	21.8	7.5	21
	***Bad, very bad***	11.7	17.3	14.7	2.4	5	2.5
**Limitations due health status**	***Severely limited***	8.1	9.1	8.6	2	7.5	2.2
	***Limited, but not severely***	28.5	36	32.5	44.7	42.5	44.6
	***Not limited***	63.4	54.9	58.9	53.3	50	53.2
**BMI**	***Underweight***	1.5	4.9	3.2	0.5	2.5	0.6
	***Normal weight***	37.7	45.2	41.4	23	60	25
	***Overweight***	39.4	31.1	35.2	47	20	45.7
	***Obese***	21.5	18.9	20.2	29.5	17.5	28.7
**Smoking**	***Regularly***	33	22	27	31	25	30.9
	***Occasionally***	4	14	18	35.75	35	35.6
	***Quitted***	23	4	5	33	40	33.3
	***Never smoked***	40	60	50	0.25	0	0.2
**Alcohol consumption**	***Consumes often, in great quantity***	nd	nd	4	6,5	0	6.1
	***Consumes in modest quantity***	nd	nd	15	51	47,5	50.7
	***Consumes rarely***	nd	nd	44	27,5	37,5	28
	***No alcohol consumption***	nd	nd	37	15	15	15.2

## Conclusions

As a final conclusion, we may state that ambulance service in the prehospital care has a great (mainly negative) influence on ambulance workers’ objective and subjective health status. Years of labour and the growing number of secondary jobs necessary because of the difficult economic environment influence the workers’ health status negatively. Shift work and the increased pace of work may determine nutritional habits and free time activities of the workers, which is mostly apparent in the chronic level of their BMI-s, which can easily be the starting point of metabolic- or cardiovascular diseases. Protective effect of physical activity is worth mentioning, thus it is an important future aim to get more ambulance workers do sports, maybe even among the collective of the ambulance station. We plan to extend our research with the current methodology nationwide, and we would strongly recommend a regular, periodic health status assessment of ambulance workers (maybe even in a well organized manner), similarly to the assessments of the general population. After a nationwide study we definitely want to assess the health status of administrative rescue workers, as well as an assessment with objective parameters among rescue workers (laboratory tests and physical/endurance assessment).

### Limitations

Our findings should be interpreted within the context of some limitations. Our literature search was performed by the university provided electronic databases, a fact that may have excluded us from identifying all studies on this topic. Nevertheless, the chosen databases are among the most adequate and representative sources in the field of psychometric measurement of mental health. Further, this study has a cross-sectional design that relied on self-report data. A longitudinal cohort study may be more appropriate to examine the relationship among different aspects of perceived health [36]. The research tool we have choosen has a number of advantages and drawbacks. The SF36 questionnaire is easy to understand, easy to use, can be filled quickly. It measures well the functional status and mental well-being as well. Suitable for assessment of healthy and sick populations over the age of 14. Also suitable for international comparisons as well since it has been standardized more than 50 languages (www.sf-36.org). It measures self-reported health, so subjectivity can be an advantage, but the lack of objectivity is the drawback. Knowing this maybe the respondents incorrectly considered their actual health status better than the general population (they do not dare to admit the truth). Despite these limitations, we were able to demonstrate that ambulance personnel in general have a worse vitality and secondary job deteriorates their self-perceived health. These results indicate the need for improving healthier work place strategies.

## Abbreviations

BMI: Body-mass index
EHIS: European health interview survey
HNAS: Hungarian National Ambulance Service Hungarian Central Statistical Office (HCSO)
IQOLA: International quality of life assessment project
PTSD: Posttraumatic stress disorder
SF-36: Short form-36 health survey questionnaire
